# Supplementary material for: Interference with the retinoic acid signalling pathway inhibits the initiation of teeth and caudal primary scales in the small-spotted catshark Scyliorhinus canicula
Source: PeerJ. 2023 Sep 6;11:e15896. doi: 10.7717/peerj.15896 (PMC10492535; doi:10.7717/peerj.15896)
Supplement: Supplemental Information 1 [file peerj-11-15896-s001.docx]

ATG codon start

TAA codon stop

**Primers for amplification**

PSPORT I inserted target gene sequence for RNA probe synthesis

>aldh1a1

ATCTCGAGAATTAGTTCTAATTAACTGCGGGCAAT**TGCTCGAAACATCCCCTTGG**ATGAGCAATACCGTTTGTACACTGGATGGCAAGAAAAGTAACGTGTATATATAGGGTTTGTGTACACATGTTAGTTTCAAACTTCATCCAGAAAGAGCACCAAATGTCAGCTGTAGAATTTGAAACCACCGGAACTGGGACTGCAATGCCTGTTTGCTACGGAACACAAGCAAACAAGCACAACATGAGTGCAGACCAGGATAGTCCTGCTCCAGCTTCCTGCAATGTGCCCGTGCCAGTTCCCGAGGTGGAAATCAAACACACCAAGATCTTTATAAATAACGACTGGCACAGCTCCAGCAGCGGCAAGAAATTCGCAACCTGTAACCCTGCCACGGGAGAGAAGATCTGCGATGTGGAAGAAGGCGACAAAATGGAAATTGACAAAGCAGTAAAAGCTGCCAGGGACGCTTTTCAGATTGGATCACCATGGCGCAGGATGGACGCCTCACAAAGAGGGAAGCTTTTGAATAAACTTGCTGATTTGATGGAACGGGACCGTGTAATATTAAGTACAATAGAATCTATTGACTCTGGAAAACTCTTCCTTCATGCATACTTTGTTGACTTAGATGGTTCAATTAAAACACTGCGTTACTATGCTGGCTGGGCTGACAAAATCCAAGGACGCACCATCCCTGTGGATGGAGATTATCTCACATTTACAAGACACGAACCAATTGGAGTGTGTGGACAAATTATACCTTGGAACTTCCCACTGCTGATGTTTACTTGGAAACTTGCTCCTGCACTGTGCTGTGGCAACACAATAGTTATCAAACCAGCTGAG**CAGACTCCACTCACTGCACT**CTACATGGGAGCTTTAATTAAAGAGGCAGGATTTCCTCCTGGAGTGGTGAATATTGTACCAGGATATGGATGCACAGCAGGAGCAACTATAACCAGTCATATGGACATAGATAAAGTAGCCTTCACTGGTTCGACTGAGGTTGGTAAACTAATACAGGAGGCAGCAGGCAAGAGTAATCTAAAGAGAGTAACACTGGAACTTGGAGGGAAGAATCCAAACATCATATTTGCTGATGCAGACCTGGACTTTGCTATAGAACAAGCTCATCATGGTCTCTTCTTCAATCAAGGGCAGTGCTGTTTAGCAGGATCCAGAATATTTGTGGAAGAACCTGTTTACAAAGAATTTGTCTGCAAGAGCATTTTATTAGCACAGAAGCGTGTTATCGGAAATCCTTTACATGTTGCAGTCACCCATGGGCCACAGATTGGCAAAGAGCAGTACGATAAAATTCTCAGGTTAATTGAGAGTGGGAAGAAGGAAGGAGCCAAGCTGGAATGTGGAGGTTTGCCATGGGGAGATAAAGGCTTCTTCATACAGCCAACAGTTTTCTCAGAGGTTACAGATGAAATGCGCATAGCCAAGGAAGAGATTTTTGGGCCAGTGCAACAAATCATCAAGTTTAAAACAATAGATGAAGTTATCAAAAGAGCCAACAACACACATTATGGATTGGTTGCTGCAGTTTTTACCAAAGATATTAACAAAGCATTTACCGTGGCTTCAGCTTTGCAAGCTGGGACCGTGTGGGTTAATTGCTACAATGCCTTGCATGTACAGAGTCCTTTTGGTGGATTTAAAATGTCTGGAAATGGTAGAGAAATGGGAGAATATGGTCTCCAGGAGTACACCGAGATTAAGACCATCACCATCAAAATTCCACAGAAGAATTGCTAAAATATTCTAAGTTAAAATGCGTTGTCTGAAGAAGTAGAAAAAGACTAAAAACCTCATAGGCTGAGCTGTATCTAAAGTTGACAATCATGCATTATGATTGATCTGTGTAGTTTCAGAATAATGCAGGTCTGTAGCTTACAGCTGCTTTGCCCCAAGAATTATAAATAATATATCAAAATAGTATTTACCTGTTAGTGTTTATACAGCAAAATAGTGATTAAAAAAACTATTTAGATTTACTAATGCTTAGTTTGAATTTTTTTGTTTCTAATGTTTGGGGTTTTAGTCAATTAATAAAAGGCTCAACTCATCTCACTGTGTCAAAGGAAGTGACAGAGTTTCCATTTTTTGTTTGATGCTGTAATATTTACCTAATTTTGCAAGTCTGTCAAAACTGCTCTCAAATTCATTTATTCATCAATTTATTTGAAATGTCAGTTCATATCCTGCAGATTGTAAAAAAAAACATTAAAATGTGTTCACACTGTTTTTACCAATTATTTTTTAACAGACAAAGGTTATGTAAATAGTATGCATGACATCAGGATGAATTTAATAAAAAGGTAAATGCATTAGTTTCAGACAACTGAGCAATTAAGGGGCCATAGCAGCTCTAACCACCAACATGAAATTCTCAACTAGAATGGTGAGGATCGGCACTGCAATTGAACCACATTAGAACCAAAAGATTCCACTTATGTCTTAAACCCTTCATAAGAAAAAGACTTGCATGTATATGGCTACTTATCACAACTTTTGGAAGCACTTCAC

>aldh1a2

CATTAATACAATCGACACAGCGCGTCTCGACAAATCGATAGGATGACTTCCAGTGAAATCGAGTTCCCCACTGAGGTGAAGGCAGACCCCGCCGCTCTCATCGCTTCCCTTCAACTCCTG**CCGTCTCCAACAGCGAATCT**GGAAATCCAGCACACTAAGCTTTTTATTAACAATGAGTGGCATCATTCAATGAGTGGGAAGACTTTCTCCACCTGCAACCCTTCCACGGGAGAGAAAATCTGTGATGTTCAAGAAGCTGACAAGGCTGATGTGGACAAGGCGGTACAGGCTGCACGGTTGGCCTTTTCCCCGGGCTCCGTGTGGAGGAGAATGGATGCATCAGAGAGGGGTCGGCTGATCGATAAACTGGCAGATCTAGTAGAACGGGACCGACCACAGCTTGCAACACTTGAATCTCTAGATAGTGGAAAGCCATTTCTCCAGGCTTATTATGTTGACCTACAGGGAGTCATAAAAACTCTCAGATACTTTGCTGGTTGGACTGACAAGATTCATGGCAAGACAATTCCAGTCGATGGAGATTATTTCACATTTACAAGACATGAGCCAATAGGGGTGTGTGGACAGATCATCCCTTGGAACTTTCCTCTCCTCATGTTTGCCTGGAAGATAGTCCCGGCCTTGTGCTGTGGAAACACAGTCGTCATCAAACCAGCAGAGCAAACGCCCCTGACTGCCCTCTACATGGGAGAACTCATCAAAGAGGTTGGGTTTCCTCCGGGCGTGGTCAACATTGTGCCTGGGTATGGCGTGACGGCTGGGGCAGCAATAGCCTCTCACATGGACATCGACAAAATCGCATTCACTGGATCAAATGAGGTTGGAAAATTAATCCAGGAAGCAGCTGGAAAGAGTAATCTGAAGAGAGTAACACTGGAGC**TTGGAGGAAAGAGCCCGAAC**ATTATTTTTGCAGATGCTGATTTGGATTATGCTGTGGAACAGGCTCACCAGGGTGTATTCTTCAACCAAGGCCAGTGCTGCACTGCAGGCTCCCGGATCTATGTAGAGGAGCCAATCTACGATGAGTTTGTCAGGAAGAGTACAGAACGTGCTCAGAGGCGAACAGTAGGAAACCCTTTTGATCCTGCCACTGAGCAGGGCCCACAAATTGACAAACTACAATGTGATAAAGTCCTTGAACTTGTACTAAGTGGCATAGCTGAAGGAGCAAAGCTTGAATGTGGTGGCAAATCCCTTGGGAAGAAAGGATTCTTCATTGAACCCACTGTATTTTCAAATGTCACGGATGACATGCGGATAGCAAAGGAAGAGATTTTTGGCCCAGTTCAACCAATTATTAAGTTTAAAACTATGGAAGAGGTAGTGGAAAGGGCCAATAATTCTCATTTTGGCCTGGTAGCTGCCGTCTTCACCAAGGATATCGATAAAGCGTTAACAGTTTCTGCTGCTATGCAGGCTGGGACAGTCTGGATTAATTGTTACAATGCCTTAAATGTCCAGAGTCCCTTTGGTGGATTCAAAATGTCTGGCAATGGTCGAGAAATGGGCGAGTACGGGCTAAAGGAATACACCGAAATCAAGACTGTAACTGTCCGAATTCAACAGAAGAATTCTTAAAGAGTGCTGTGAGGCTGGTGCTTTGTTCATGGACCCAGTAGGAATCTGCTCTGTTATTGACAGAACATAATGCACAACAGTGGTTGTGCAGCATACGGTTGAAGCTTTATCTTATAACAAAAAAGTAACAAAAAATATACTAGTGAATGTATATGTACCACAAATACTGTGTATTTGAGGAAAATACAATATAATCTCAGCAACTTCCCCGAGTGCATTCATTACATGGTGCTCCATCGTAAATAGTGTTTAGCCTTTGTGTAAATCTGTCAGCTGTGGAAATTTAATGCACCATGTATACTCAATGGTGTCAATGGAACTATGACAGCCCAGTTCTGTCGCTACTAACAGTTTGGGAACTGCCACTACCACACTTTTTCTTTTAAACCAGCGTGTCCCACTGGCCGGTGCTGAGCAGAGGTAAGTGGAGCAGGCTCACGTTGAAAGGATGAGGGAGGCCAGACTGATGTTAGTGTCCTCTCTTCCTGGAAAGGGGCACAGATCGGAAACATGAGCCCTGGCCCACGCACTGTAACCCGAATATAAATATTTAGGTATTTGCTCCATGGTTTGTTGGTAGTACGCCGATCATTTCTCACAACCACTGCCAGCGCTGTAAAAGAACCCAGAAACTGCTGCAAGAGCAGGTTTCAAGCTGGGAATCCAACTTTTAAAATAGATATCTGTATATGCACACCGCTAGCACAATTTTAACATGAAACTTTCATTGTATTCCACTATGTACAAGGGAGCCTTGGAGTTCCTGCAGCCAGGGAGGTTATTATTGTGGGGGTGGGGGTGAGGGGGGGTGGAAACTTGATTCCCCTTTCGATTTGACGGAAACACCAGGAGCTTCCGTGGGCCCTTCCAGCAGAAGGTTCAATTCTGACCTTATGCCAGAAATACCTGTGAATCCCTGTTAGTCCAATTTGAGGTTTGAATGCCTACCATCTAATGCAGTTGTTGCTCGCAAACCCTTTGATCGGAGTAGCATTGTAACATTGTTACAGGACAGAAACAAGCTCCTCCGGCCCAACAGCTCCATGATTGTGTTGATGCTCCACCTGACCCTCCTCGAACACCACTTCCTCCAATTCCACCTCCTGTTCCCATCTACTTCCTGTGTTAATCTAGCTTCCCCCTTAAATGTACCAATGCTAGTCAACACAAGCTACTCCCTGGGGTAGGGAATTCCACATTCTCATCCAAAGATGTTTCTCCTGAATTCCTGATTGGACTTATCAATTACTATTTTACATTTATGGTGACTCGTTCTGGCCTCACCCACAAGTGGAAATATCTTCTCCACGTCTACCCTATCAAACCCTTTCATAAATGTAAAGGTCTCTATGAGGTCTTCTCTCAGTCTCCCCTGTTCCAGGGGAAAGAGCCCAATCTGTTCATTTTTTTCCCCCTGTTGCAGCCCGATACTCCCACTATTCTGCCCAGTCAGGTGGGGACCTCAGATGTGTCAGAAGCAGAGTGGGCACCCACTCATAATATGCAAAGAGCCAAGTCCCAGTATTTGCACAGGGGTCAGTGACAGTCTGCACAATGCCTACTCCCAACCTCAGGCCACCCCAGGATATCCAGGACCCGGATCCAATTGTGTTGGGTAACAGAAAACAATGTTAGACTATCTAGCCCCCAACATATGCAAGAGGAACAGAATAAACTCTGTAGTCGGGGGTTGGGGGGTTTGTCATTAGCACCAAGTTCAAAGCATCTTAAACCTGCCAGTGCTCTATCCAATGAGGATAAGCTTTCTCCTGTCATTATGTGCAGTGACCAAGAACCTAAACACTGAAGATTTTCCTCTGGTTGTTATTTCTAGCAAGGTTTACACTTTTTGAACATTATAAAGAATTCTCCAGGAATACCTTCTCGGACTGCATTTGTTTATGTCTCAATGGGTACAGAAGCCAACCCACGGATTGCCTCGACAAAACCAGGCTTCCATAACTCATGGGACACTAACTTTTCAGCAAACCTTTGTTCTCACTGATTTCCAAAGACAGGTCGGAAAATTACAATGAACAAACAGCGCTCTACTGAAAACAGCATATTGCCTCGACCTAGGATACCTACTCCCTTGCCAAAGGAAACAAGATTTGATAAAAAATGTCTTTACAAAGACAAAGCCCGTATAAATGTGCCATGTCCTCTTTCTATGTTGTCTGGGACATTGTTACATGTTGCGCCATTAACTGTTGAGTGTTATCAATGTATGGAATAATTCTTCGGACGTAGCAACATTGAGAAATTCAATGTTTAGGATACATTGTTTAATGGCAATGTTACAATGTAACACAAGTCACTTGGCACTGTGCCAGACATTAGGCATCAATGTAATAATTTGGAATATTTTTGACCAGAGGTTTCAATACCTGTCCTAATAAATCGTTACAATTCTTAACAACACAGATGACGGCTCAGCAATCTGCAGCACATATCGGAGAAATAAAACCTCTTAATCAGATCTGTTGGCAATAAAATCAAAGAAGGTCTGATATGAAATGGGAAGATACCAAAGTAATTAGAGGCATGTTTCAGTTCTGAATATTTTCTAGTGATTACTATTAGTGATAATAGCAGTCTAACAATGAACACACTTATAGGGCATAGCTCTGTCCGGTGGACTTTACTCCATTTAAATTAGAAAGTTAACACTTATATCAAAATAATAGGTAGAGGAATTTCATACAAAGTCATTAAGCATTCATAAGTTAATATCACAGTCATTTCAAGGCTTTTGGATACGTAAAAGATTTTGTCATTACACTATACTGACAGTGATAAAAGCTAATCCTTTACTATACTTCACCCTTCTGACTCCACTCCTATGAAATCTGAGCATTAATTTGATAAACTTTCCCTATTCCTGCTGTCTTTCAGATTCTCTGATTCAAGTCACCTCTGGTTGATGTCACAATAAAAAGCAATAATGTATAAATACTCGCATTTTTATACACTGATTGAATGACTGCAAATTTCTTCTTGTTAACTGTACTGCAGTTTTGAAATCATGTATATACAATTATGTACTATGAAGATATAAAGATATTTTCTATACTGTTTTTCATTAAAGAACAAGACACATTTGGTATTGGAGTGTGAAAGTTATGTAAAACAATTTGACTAAACACCTGCTGATGTTTTAGATCAGAGTATTGGGCGCGTCGTGTTGGTTGCGGCTAGATCAGTACAAACAAAATCCCT

>aldh1a3

CCCTGACTTTCAGC**ACCATTCATGCTGCCACTCT**TTTGGATTTATTAATTGACTGGGTTAACATGAACTCCACGGTCCAGATTAACTCAACAATAACACATCCACTGTGTGGAACCACAGTCACGGGAAACACTGGCTCAGCGATGGCAGAGAACGGGGTGATGGAAAACGGAGAGACAGAGAGAGAATTGTTGCATCGGCTGCCGAATTTCCCCAAAAACCTGACAGTGAAATACACCAAGATCTTCATCAACAATGAATGGCATGAATCAGTCAGCAGAAAGAAATTTGCTACATTCAACCCAGCAACAGGGGAGAGAATTTGTGATGTGGAGGAAGGCGATAAGCCAGATGTGGACAAAGCAGTAGCAGCTGCAAGAGTAGCATTCCAGCAAGGCTCCCCGTGGAGAAATCTGGACGCATCGGCCAGGGGAAGGCTCTTATGTAAATGGGCCGACCTCATTGAGCGAGACCGGCATATTCTGGCTACATTGGAAACTATGGACACAGGAAAACCCTTTCTGCAATCATTTGCCATTGACTTAGAGGGCTGTATAAGAACGTTACGTTACTACGCTGGCTGGACTGATAAGATCCATGGAAAAAGTATACCAGTGGATGGAGATTTTGTGATGTTTACAAGACATGAACCTATTGGCGTGTGTGGACAGATTACACCATGGAACTTCCCCCTGTTGATGTTCATTTGGAAAGTGGCACCTGCACTTTGCTGTGGGAATACAGTGGTGATCAAACCTGCTGAACA**GACACCCCTCACAGCACTTT**ACATGGGATCCTTAATCAAGGAGATCCCAATCCCCCCAGGTGTGGTGAATATTGTCCCCGGATTTGGACCCACTGCCGGGGCAGCGATCGCGTGTCACAAGGACATTGATAAAGTTGCATTCACTGGTTCCACTGAGGTTGGAAAGTCCATCAGTGAAGCAGCCTCCAAAAGTAACCTGAAGAGATTGACGCTGGAGCTGGGCGGGAAGAACCCCTGCATTGTGTTTGCTGACTCTGACTTGTCCCTGGCAGTTGACTGTGCTTCACAGGGAGCTTTCTTCAACCAAGGCCAGTGTTGCACAGCCGCTGCTCGAATATTCGTTGAGGAACCAATTTACGAGGAGTTTGTTCGCCGCAGTGTGGAACGGGTCAAAAAACGAGTGGTTGGAAATCCTTTCGATCCGAAAACCGAACAAGGGCCTCAGATTGACCAGGTTCAGTTTGACAAAATTATGGAGCTAATAGAGAGTGGCAAGAAGGAAGGAGCCAAGTTGGAGGCTGGCGGATGTGCCATTGGCGATGTGGGTCATTATATTCAGCCGACTGTGTTCTCCGAGGTGACCGACAACATGCGCATCGCTAAAGAGGAGATCTTTGGACCAGTGCAAGTGATAATGAAGTTTAAAAGCACAGAGGAGGTTATTCGAAGAGCCAATGATAGTGAGTTTGGACTGACAGCTGGAGTATTCACCAGAAATATCGACAAAGCGTTGACTTTGGCTTCTGCGCTTCAGTCTGGGACAGTTTGGATTAATTGCTATAATGCTCTTCATGCTCAGGCGCCTTTCGGGGGCTTCAAAATGTCTGGAAATGGAAGAGAACTGGGTGAATATGGACTAACAGAATACACGGAAGTGAAAACCGTCACTATCAAAATCTCACAGAATAACCATTAA

>cyp26a1

GGCTTGAATCTGGAGTGTGAGCATTTGGGGAGACTCGCTCGCCTCACTGACAGCACAGTGCCACTGACTGACTTCTAGGCAAGGGACACTGAAAAAAGTACTGCCGCTAAAATGGTCTTATTCACACTTTGTGCTACTTTCCTGTGCACTTTCGTGTTGCCCTTGGCGCTCTTCCTCGCTGCCCTGAAGCTGTGGGAGGTGTATTGCCTGAGCAGAAAGGACCCTAGCTGTGAAAAGCCCCTACCTCCGGGCTCCATGGGGCTGCCCTTCCTGGGAGAGACTCTCCAGCTGGTCCTACAGAGATACACTTTCCTGAAAATGAAAGTGCGGAAATACGGCCACATCTACAAGACTCACCTCTTCGGAAACCCCACCGTGCGGATCATGGGAGCGGAGAACGTGAAGCAGATTTTGCTGGGGGAGCACAAGTTGGTGTCGGTGCAGTGGCCCGCCTCAGTCAGGGCCATCCTGGGAGCAGGCTGCCTGTCCAGCCTCCACCACACCGAGCACAAGCACAGGAAGAGGGTGATTTTGAAAGCTTTCTCCCGGGAAGCTTTAGAAAACTACATCCCGGTGATGGGCGAGGAGATCAGGGCCGGGCTCAGGCGCTGGTTGGAGAGCGGTTCCTGTGTGCTGGTCTACCCCGAGATAAAGCGCCTGATGTTCGGCATCGCCATGAGGATTCTGCTGGGCTTCGAGCCGACACACACCACCCGCCAGACCCAACAGGAACTCATCGAGGCCTTCGAGGAGATGATTCGCAACCTCTTCTCCCTGCCCATCGATGTGCCCTTCAGCGGCTTATACCGGGGGCTGAAGGCGAGAAATGTGATCCACGCCAAAATCGAAGAGAACATTCGGAAAAAGGTGGCGAGACGCGAGGCGACG**TCTCAGTTCCAGGATGCTCTG**CAACTGTTAATTGAACACTCGGAGAAGAGTGAGCAACCCCTGCGTTTACAGGAGCTGAAAGAATCTGCCACCGAGCTGCTGTTTGGTGGACATGACACGACAGCCAGTACAGCTACCTCCCTGGTCACTTTCCTGGCCTTACACCCGGAGGTGCTGCAAAAAGTCAGAGGGGAATTGCAAGAGCAGGGATTACTGAGCAGCGACTCTGCAGAAAACAAACAAATCACCATTGAACTCCTGGAGCAACTTAAATATACCGGAAGTGTCATCAAAGAAACTCTTCGACTGAGTCCCCCGGTCCCTGGAGGATTCCGCGTGGCCCTAAAGACCTTCGTATTAAACGGATATCAGATACCAAAAGGCTGGAATGTCATTTACAGTATCTGTGACACACATGACGTGGCAACAAATTTCTCAAAAAAGGACGAGTTTAACCCAGATCGCTTCATGGAGGGCTGCTCAGAAAAAGATGCCTGCCGATTTAGCTACATCCCATTCGGAGGTGGCTCCAGGAGCTGTGTGGGCAAAGAGTTTGCCAAGATCCTCCTCAAGATCTTCACAATGGAGCTCGCCAGGGCTTGTGACTGGGAACTTTTGAATGGGCCTCCCATGATGAAAACCAACCC**TACTGTATACCCAGTGGACAA**CTTGCCCACAAAATTCACACCCTTCCGCAACAAAATGTGACCTATTCGTAACACACCCAGAACACTGCAGATCACCATCTCAATGTTATTGTGATCCAGACTTTGTTTAAATAAAGTTCACAGATCATTTACTTATTGTATTTGAGCCACGAGACCAAGAGGTCAAAAGATCTTTCAGGAACTCACTCGGTGGATTGACATATAATGCACAGTGGGCTAAACAGCTGGC

>cyp26b1

CATCTGATTCAATTTCCAGATCTTTGCCAAGGTTTTCAGCCATGATGCCCTGGAATGTTACCTTCCCAAGATCCAGCAAGCCATCCAGGATGGTATCCGAGACTGGAGCAGCAGTGTGGAACCCATCACAGTATACCAGGAGGCCAAGAAACTGACATTCCGCATTGCAGTCCGGGTCTTACTGGGATTCCGCATTTCCGAGACTGAGCTGGACATCCTGTCAGAGAGTTTTGAACAACTTGTTGCCAATCTCTTCTCCTTGCCTCTCGACTTTCCATTTAGTGGTTACAGGAAGGGTATGCAAGCCCGGGACAACCTTCATAAATACCTGGAAAAAGCCATCAGCGAGAAGCTACAGAGCAAACAGGATAAAGATTACTCAGATGCGTTGGACATTCTGATAGACAGCGCCAGGGAGCACGGGAAAGAGCTCACCATGCAGGAACTGAAGGAATCCACAGTGGAGCTGATCTTTGCCGCCTTCGCGACCACCTCTAGCGCGACGACATCGCTGATCCTCCAGCTCCTGCAGCATCCAGCGGTACTAGAGAAACTGAAGGAGGAGCTGAGGCATCATGGGATCCTTCACAATGGCTGCAAGTGCGAGGAAACCCCCAGGATGGCCACGGTGGTGCGCCTCAAGTACCTGGACTGCGTCATCAAGGAGGTCCTGCGTTTGCTTCCGCCTGTCTCAGGCGGCTACCGGACAGCA**CTGCAGACGTTCGAGTTGGA**CGGGTGCCAGATTCCCAAAGGCTGGAGCGTCCTCTACAGCATCCGGGACACCCACGACACGGCTCCCGTGTTCCAGAACGTTGAGGTCTTTGACCCTGACCGCTTCGGAGAAGATCGGGACGAGAACAAAGGCGACCGGTTCAGTTACATCCCTTTCGGAGGGGGGATCAGGAGCTGCCTGGGCAGAGAGCTGGCCAAGCTCATCCTAAAGGTGCTGGCGATGGAGCTGGCCAGCACCAGCCGGTTCGAGCTGGCCACCAGGACTTTCCCCCGCATGTTGACGGTGCCAGTTGTCCACCCTGTGGACGGGTTGAAAGTCAAATTCTCTGGACTCGATGCCAACCAGAACGAGATAGACCCCGAGACAGAGTCAATGCTGGGCTCCACGGTGTAACGGGGGTGGAGGGGCCACTCCAAAATCCCCGCCACCTCTCACCAAACCCCACTGAAGCAACAACAATAAAGCAAACACACCTGAACTTAAAAAAAAAAGAAAACTCTTTGATAAACTAATGTACAAGGTAGAAAGACGAAACGGCCCCCTCTGGGAAAACCCGCTCACTGAATTTCCGTTGTGGTCTGTCGTAAGGGAGGTGATTTTA**GACGGGAGAAATTCACGGGA**GAGTCGCAAAGAGAGAATTCCGACTGAGAAGGTAAAGAGTGATAATTCGGGACAAATGGAAGAGATGGGGAGCGGCGGAGGAAGAGAAGAGGATTTGACCAACTCAAGTTGGGGGGGAGAGAGGGAGAGAGAGAGAGAGAAAGAAGAGGAAAAGATATCCGAAGAAAGTTGGTGGAAGAGAAAACCTGTGAGATCAACGGAAGACAGAAGACTATCTTTAAAAACAAACACATATATATCCTGCGTTCCTACTTAAAGTATATATATGTATAAACCTACTCTGAGTACCATTCTAGGTATATAACTTGATTAGAGGTTGCAACAGAGTTACAGCCTTTGCACTTTGAATGTTGATCCAATTACTGATGCTGGTTATATTCAGCGCAAACTTAGACACCCTAGCAAACTGGTTTAAATTTGTACTATTCCTATCTTTAAGCTATATATGGATTATTTGTCGTGTGTTTTTTATTTCAAGATTGCAATTAGACGTCTAGTGTAAAAAGAAACTATTTTTTTTCTGTTGGTAAATATTGTTTTAGTAAAAAATGAACAAAAGATTCCTTTA

>cyp26c1

GTGAATCAGCTCTATACGACTCACTATNAGGGAAAGCTGGTACGCCTGCAGGTACCGGTCCGGAATTCCCGGGTCGACCCACGCGTCCGCCCACGCGTCCGGGATGCTGGCCCGGGTGTTCAGCCGCGGCGCTCTGGACAACTACATTCCTGGTATCCAGCAACTGGTCCGCAGCCGGGTACGGGGCTGGTGCAGGGAACGGGCTCCAATCGCCGTCTACCCCGCCAGCAAATCGCTGACTTTCTGCATCGCCACCCGGATCCTGCTGGGCCTCCGGACGGCGGATCAGCACCTGGACCTTCTGTCGGGCACCTTCGAGCAGCTGATGGACAATATCTTCTCGCTGCCTGTGGACCTCCCGTTCAGCGGGCTGCGGAAGGGCATCAAAGCACGTAACGTACTGCACGAGTACCTGGAGAAAGCAATCACAGAAAAACTCCAGGCAAGCGGGGCTCAAGCGTCTCCCGATGCTTTGGATTTGATGTTGAACAGCGCCAGAGAGAACGGGAGGGAACTCAGCCTGCAGGAGCTGAAGGAAGCTGCCATTGAATTAATCTTCGCCGCTTTCTCCACCACAGCCAGTGCTTGCACCTCCCTGGTCCTCTTGCTTCTCCAGCACTCCCAGACCCTAGAGAAGGTGAGACAGGAGCTGGAACAGCACGGGTTGATTCGCCATTGTCAATGCCATCATCCCATCGCATCCCGGGATAACCCCGCTCCCCAGCAGGTCGGTCAGGCCGATAA**CAAGGCGACTCAGGACATCGA**GGGAGCTCAGCCGGGACGAATAACCCGAGACCAGCAGACTCGGGCTGAAGGCTTCCACCAGCCCCTCGTTCACCGAGAGGATTTAACCCGGGACAACGGCTGGCTTCCATCCACTGCCCCCACTGGAAATCGAACCCAGAAAAAAGAATGCCACCAAACCGGGTCACCCTGCGTGCCAAATCCAGGGTGGAAGCAGTCGTCAGTCCCCGTTCACTGCAGACAGTGAGATGTCCGGAGCCGCAGTGACAGGCAGTGAGGCGGCTCAGATGTCCCAGGATTGTGACTGCCAACACAGTCTGAGTGTGGAGTCACTCAGCCGCCTGCGCTATCTCKACTGTGTGGTCAAGGAGGTGCTGAGGTTACTGCCGCCAGTGTCTGGGGGCTACAGGACAGCGCTCCAGACCTTTGASCTGAATGGGTGTCAGATTCCCAWWKKYTGGAATATTATCTACAGCATTCGTGACACGCAGGAGACAGCGGCCGTGTACCTGAACCCGGACACTTTCGACCCTGAYCGCTTTGGACCCGAGCGGGACGAGAGCAAAGCGAGCAGGTTTAATTATTTACCTTTCGGCGGGGGCGTCCGGAGCTGTATAGGGAAAGAGCTGGCGCAAGTGATCCTTAAAACGCTGGCCATTGA**ACTGAGCACAACAGCGCGCT**GGGAGCTGGCGAGCAGCACCTTCCCCAAGATGCAAACTGTCCCGGTGGTGCATCCTGTCGATGGGCTGAAGGTTCGCTTTCATCGCTTGGACGGAAAGGAGGAGAACGTGCCCATAAGAGAATGACCTCTCTCTCTCTCTAAATGTATAAATATATATAAATATGTATAAAACAAGGCACTAAGTGCAAGCGAAAATTGGCTGCTGACCACTTCCCGCGACTACCAACAACGTGGCCAGTTTAATGCAGACTATTGTAAATGCCAAACACGTGTGAGACCCACAGTTCGGCAAAATGCCCAGTCAATTGATTAGAGAGGCGAATAACCCATTTTCTAACAGGTATGGTCGAATATTGTTTGTTTCAGAATAACGTTGGAAAACCCGCGATTCTGAGGAAATTCATGCCAATCATTCTCTTCATTTGAACAGAACTGGCCAGTTTTCTTGAAGCTGTGGTTAGAAGCTCAAGTTTCGGGCAGTTCCAACACTGAGATCATGAGAATGAATTCCTCCTTACTGTGATAGTCAGTATTTCTGCTAGGTTTTACCGAGGAAACGGGTGTTTTTGTAAAGGGAACTTTTACCAGATGCCTTCTTGTTAGTTCAGCCCCCCCTCYCCCCCARATGCAGATGTGTTCATCTTTCACCCAGSGTGGAAGCTGTATGGATACMCTCAAGTAAGTATCSCTCGGTTAGTCAAAMAGTTCCATTYYTATGAAGTKKKGTGAAGAMAAGCTKKGAACMGCC

>rara

TGTCCTTAGGAAGCAGAGGTGGAGCATAGCACAGGATGTGCAGATTAATGGCAGATTCTGAAGAGTTGGTGAAGCAGATTTCCTTCCTCGGCCTGTGTCGGTCTAAACAGTGATGCGGCTGTAGTTTTCTTTGCTTTTTTAAAATCAACTAGCGAGTGATTTAGGTGTGAAGGTTTCAGCTTTGGACACATTTCTTTATTCAGCAAGTCTCTGAGGCCTGTGAAGGTACCCTGTTTGACTTCGTTTTGGAACACTTGGGGATGCAGTAAATATTTCAGTGTGTAAGCACACCCAGTCTGGCCCAGATTTAGAGGAGAAGAAGGCCTATTCAAGGAGTCTCTGAGGACGTGAAGAGCATTATTCCTACAAGAGTTCAGCTGCAATATACATTAGGATAGTACTTGGATAGATTCATATGGAACCAATGGTTGACTACCAGGCTTGCCATCCATAAGGAATACAGACGGAATTTTTAATTTTTCTATTAGGAGAACTGGTGTTACATCATTTTTTATATGAACCCTGCTTGTTTCTGAATTCAATTCTGCACGTCAAGGCTGTCATTGCCTTTGCTTTCTCTGGACGTTTCTCCTATTTGATATGCACTGATCAGCTTTGTGGTGCACATGTGAAGACTATCCTGTACACGTCTTACTTCACCATGTC**AACCAACAGCAGTTCGTGCA**CACCGCCGGGTAATGGACACGTAAATGGTTACCCAGTGCCCCATTACCCATTCTTCTTCCCACCGGTCCTCGGGGGCCTTTCACCACCATCCATAAATGGATTGCAACACCAGCTTCCAGTCAGTGGCTACAGTACCCCCTCACCTGCCACCATTGAAACACAGAGTACTAGCTCAGAGGAGATTGTGCCCAGTCCACCTTCCCCACCCCCTCCTCCACGTGTATACAAACCCTGTTTCGTCTGCCAAGACAAGTCATCAGGCTACCACTATGGAGTCAGCGCTTGCGAAGGATGCAAGGGCTTTTTCCGAAGAAGTATCCAAAAGAATATGGTGTACACATGCCACAGAGACAAGAACTGCATCATCAACAAAGTCACTCGAAATCGATGCCAGTACTGCCGGCTGCAGAAGTGCTTGGAAGTTGGCATGTCCAAGGAATCTGTGCGAAATGACAGGAACAAGAAGAAGAAGGACACCCCCAAGCAGGAGTGTTCAGAAAGCTACACAATCACACCCGAGACTGAGGATCTAATAGAGAAAGTACGCAAAGCCCATCAGGAAACCTTTCCAGCTCTGTGTCAGCTTGGCAAATACACTACTAATAACAGCGCTGAACAGCGAGTAACGTTAGACATTGACCTCTGGGACAAGTTCAGCGAACTCTCGACTAAGTGCATTATCAAGACAGTCGAGTTTGCCAAACACCTGCCTGGGTTTACCACTCTAACCATCGCTGACCAAATCACCCTTCTCAAAGCTGCCTGCCTAGACATCTTGATTCTGCGAATCTGTACCAGGTACACCCCTGACCAGGACACCATGACTTTCTCTG**ATGGCCTTACGTTGAACAGG**ACACAGATGCATAATGCAGGCTTTGGCCCCCTCACTGATCTGGTTTTTGCGTTTGCCAATCAATTACTGCCATTGGAGATGGACGATGCAGAGACTGGGCTTCTTAGTGCCATCTGCCTCATATGTGGAGATCGTCAGGACCTGGAGGAGCCAGAGAAAGTGGACAAGCTGCAGGAGCCGTTATTGGAAGCCCTGAAGATCTATGTGAGGAAAAGACGACCGAACAAGCCCCACATGTTCCCAAAAATGCTGATGAAGATCACAGATCTGCGCAGCATCAGTGCCAAAGGAGCTGAGCGGGTAATCACCCTCAAAATGGAGATTCCCGGATCCATGCCACCACTCATTCAGGAAATGCTGGAGAATTCCGACGGCATCGACCCTTCCTCCTCACCTTGTAGCAAAAGCAGCGCTGCCCGCCCGTGCAGCAGCCCGAGCATGTCCCCAAGCTCCGCCAGCAGCAGCCCCAATACTCAGTCACCATAGCTTCGGGCGTAAGGAGCAAGTGAGTCGGACTCTCACTCACATGGACATTTTGACGTGTTTGTAAAAGGTGAAGTGTTCACAGGAGAGCCACAGTATTGTTTGCCACATACTCTGCAGCTTGCGGAAGGCGATTGGGACTGAGTTGGAACTTAAAACACTGTGCTTAACCACGCTGGAAGCTCTTTCAAATGTGCTTAACTGAGATGGACACGAGTTCGGGCTGTGCTGAGCAGCAGAGATGAACACCACACACACACACACACATATACACCCACCCCTCTCCGAAATAGGAATGAACCATTGTTGAAGCAGGTTGTGCTTCACTTTGCAAGCCTTCAGCTTCTGGTTTTAGAACTTGCTTTGATTTAACGCTAGGAGAGAACTATGAAGACCCTTTGTAGTTTTATTTTTTGAATATTTTTCCTCTCCTTTTGTTTATCTTTCATAATCTCATATAATACTATCTGATAGACTGGGTCACCAGCGCAAAGTCCAGTGGCACTCAAACGCAAGGAATTTGAACAAAGAAAGATTTATATGAATGAAACATTTAACTGATTATTTTTTGTTGAACTGTTTATATTGGATTTTGCAGAGGAAGGAAAACAAATTATGGCTTCATTACATTTTTGAAATGAGTTGCATACAAGCTGGAAGATAATGAAGGTAACTAGGTTACACTAGGGATGTCTGTAAATATGCTGTTCTGTTTAATGTACTCACAAGAGGTTTCCAATCTTTTCTTTTATGCCTTTGTTGGTTGGATGTTCATTTTAAGCACATTTACTCTGAGAGAGCGTGGTTCTGAATTACTGTGTAGGTCTCGAATATCCCTCCCAGGCCACGGCCCGGCGAGGGAGCCGTGCATGTGTGATCGCACGCCAGGGCCATGCTTCACACGCCGCGCTGTGATTGAGATGGACTGGACTGGATTGGACGGTGCCCACTCTCTGTCTGGGACCGAGAAACGGGGTCACAATTCCAGCTCAGGGCTGCTGACTGAGAAGGTTCCCGTATCCTTGGGCTTTAATTGACTGGAGCCTGAGATTGTGCGCTACCTCCGACCGTGGTGAAGTGTATCAGTGGGGCTGGCGACAGCTTTAGCAAACAAGCCTTGCCTTGCCCTCACTGACCATACGTATCAACCTGAGAAATTATCAGTGGATTATTTTGAACTGCAGTTTGCACAGGAGGTTCCGTCACATCCAGTGGTGTCATTGGCAAAAAAAGGAATTTGAGCTTCTCAGGATTTGACCGTGATGTACTGTGAACATAGACCTGCATAGTGCTCAGTATGTTCTGATAGTTCATTCAAAAGAATGATCAATCACCTTTCGACCTCGCCCTGTTTGACTCTCAGTAATCTTGTTATAGTTAACGCTTTAAACTGCATTGGAATTTTTTGATATTTGAGGCTGCCGTGTTTGAAGCCATGCGCGTTTCTTAAGAGTGAATCAGGCCGAACTGGATACAAGTGCGCTGCCACAGGCTGAGGTGAAACGCCACTCCTTCAAAGTTCATTCTTCGTCTGCTCTCTACCTCTCTCGTCAACTTGCAAATCTCGACTCAGGTCGGGCAAACTGCTCTTCCAAGATGGCACAAACCGGCATTCTTTTATATTCCTTCTCAGAACGAGGGCATCGCTGGCAAGGCCGACATTTATCGCCCACCCCTAATTGCCCAGAGAGGGTGGTGGTGATCACTAGTGGGAGGTTCGATATGATTGAGTGGCTTATTAAAGCTGGGTCAGAAAGTAGTTAAGAGTCAGCCACATGGGTGTTGGACTGGAGCCATCGATAGGCCACATAAACACGGCAGATTTCCTTCCCTAAAGGACATGAGTTGAACCAGTTGGGTTTTTTGCAACAGTGCAACATCTTCGTGGTCACTTCTACTGGTGCCAATGTTGCATTTCCCAGATTCTTTTTTTTTTTTGGGTTCATGCTGAATTCAAATTCTCAAACCCCAGACCATTCAGGCCTTTGCTACCATTTACCGAAGCTCATGTCTGACTGGCGTTGTGCGCGCTCAGGTGATAGAAAACTGGTGCATCCCAAAACAAGCCCTAGCGATTCGGCGTATTTGAACAAGTGTAAGACAGAAAACATCAGCCGAATGGACTGAAACCATTTTCAATCGATCTGGGTTCCTCCATCAGAAAGATTGGGCCGTGAGATTGGGATGGGGGGGGGGGGGGGGGGGGGGGGGGAGAAGGGTTATTGATGAGTTTTCTCACATCAGTTGTGAGGCAATTATTTTGACCCTCAACCCTTCATCAAAATTAGAAACCCAATTGCAGGAGCAAGATCCCGCAGCTCGCGAATCTGTCCCCAGCAAAGGTCAAGGTCTTTTATTCGGTGGATGTTGAGGTCTCCGATGATGGGATTCCTTCGGTTGGTGACTGAACTTGTGACAAGATGAAAAACTAGGAGGATAAGGAAGCAAGGTCCCTGTGTTCAGTTGTGAAGGTTAAAAGGCTGTGGGGAATCATTGGGCTGTCAGTCTGGGATGAGATTCAGTCGAGGCATCATTTTTTTAACAAAAAAAAATGAATTAATTTACCTGCCAGACACCAAAACATATTTCCTTGACTGAGGAACACTGGCCTTGCATCAGCTAGACAAGGGGCGCGGTTCACAAATGGATCTGACCTGAAGTGATGCTCTGGGATCTGTTTGGCTTGAAAGATAGACTTCATGCAGATTTGTTTCCTTAATATGGGACCTCACAAACGCTTTAAAAGGAGTTCGCTTTTCCACTCATATCCCAGATTTTTGGCAGGAGTTGCAGTCAGGGTCAAGAGAATTCAGAGGCTTTCATTCCCATTGAAACAATAGAGATGAGGAGCCACCTTCTAGGGAGAAACATGGGGTGGATGGGTGGGGGGGGGAATAAGGTGGTATTACTATGTGCAAATTGACAGTCTTTCACCCTGAGCACAGCGCAGCAGCAGCAAGGTGCACAGGGAGTTCAGCATCGGTACAATTGGCAATAACTCCCGGGAGCTCCAGATAGGATTTTCAGAGCTCGCAGACTCGGCTGATGTAAGATCCGACGCGATCGATGCCTTCGTCTGGGCTTCAGTGAAGTCCCAGAATCGCCGTTGAGGTGTGGCAAGGCCGCCCCCCCCACCCTCCCCAGTCTCCTACCTCTGCTGCACACGGGCTTCACAGGGTTAAATCAATAAAAGAACGTCACAAAGTGCATCCAGATTCACACTGCGCAGGTTCCCACAGAAGGTTTGGACCATCTCATTTGGATATTTCGGCGAAGGTGCCCTTAAATTGGATACCAATTGTACAGTTCTGTAATTTCTGGTGAGCACAACACAAAGAAAGCCCTTCAGCCCACCCCCTCCCAGTTTGGTGACTGTTCCAGGATAAAAAAACACACCTTTCCGAAGGTGCAGGGTCGATGCTTTACCAGATTTGAAGGTTTTTTTTGGCGCAGGTTGTATCTTTGAGCAAATTCTCCCCCTCAAGTGGCTCCAGTTTTCGTTCAAAGGAGAATTCGCAGCTCAGTATTAATTTTTTATATATATATATAATATTTATTTGAATTTTATCTCTTGATTTGAACAAGGCGGCTTCAGCCCAAGTCTGTTTTCCCCATGCTGCGTAACCACACAACCACAGCAAACGTGAGCAAATAGTGCCACCTACTGGTTATGGTCACCATCGCAAATTAATTTTCAGTGCACGGCTCAAGTGGCCCAGCAGCAGAAACTGCTCAAAGTTGGCACCTATTTCCCAGTAACGTCTTGCAGACATTGGAATGCACAAGAGGAAGACGCCATGTATTCCTGGCTGTGCCCCAGAACCGATGGGGCAGTCATTGTGAGTTATTGCTCGTGATGAAAACCTTCCCCCCCCACCCCTGGCTGCAGTGTGGTTTCCTCTTGTTCACGGATGTTAATCTTGTCGCTCCACTTTCCTTCCAGCACGCCCCTCCCCGCACCTTCCTCCCAGCCCAGTGACAGCACATCTCTGTGTTTGTGAGTGGCAATGTGGCCCAACTCAGTGTCATATTTTACACTTTTTTTCTATTGTAAAACAGCCACCCGTGGTTTTATCAAATGGAAAGGTACAGTTACACATTTAGTTGAATCATTGACCTGACACCTCCACTGTTAATGGCGAGTTCTTGTTCTGTTCATGGATGTGTTAGTGTACCGAGTCAGACTGTACAGTAAGATTTCCTACAACAAGGCTTTCTCTGTGCAGAAGTTACTGTTGAACTTTTCATGAAGTCTACTGGGAAAAGAATAAAGTTATATTTATTCTACAATTGCACATAGTCGTCTTTGCTGATGGGGCAATTGCTGTATTCCTGGAATAATGCGTTACTTTTCTACTTGAGCGGCACCAAGAATCAGAATTCCGCTTGGATAATAAGGCTGGTTCTGGGATTAGCATACGGTGAGTG

>RARb

TGGCTTCTGGAAATATGAAGTGCTGTGGGAATCCTGGGAGCTGGTGATGTCAGGCTGGGTTGGGTCATTTGAAGGTTAGCAGCCTGGGTAGGGTTCAGAGAAAGTTCACTTGCATATATTAGGCAATTCAATCTTTTACTGTGCTTCTGTGACATAAGGAGTAGGAAGTAAGCTGGTCAAAAGCAGGACGAGCCATTTTGGAAAACAAGAGGGGTGAGCGGAGCTATTTTTGCGGCTGCAGATTTGTAACGTGCAAATTGTGCCAGGGATCGGGAGATTTCCGCGGGTCCCACAGAGCCAAGAGCGAAAGAGACTTTCAGCGAAAGGGCAGCTGCCAAGGCTGAACATTAGACAGCCTGACTCCCCCTCTCCTCCCCTTCGCCCTCTCTCATTTAGAATTGAGCGCCGTATCTGACTTTGGGGGAAAAATGGTAAATGATCATTTGGATCAATTACAGGCTTCGAGTTGTGTTGTCTGTCATAATTCATGAATCCGCTCTGGGGAACAGACCAGCAGTTCTTAACGTGCCAAGAAAAAGAGGGGCGAAAAAAAATATAAGGGCAGAGTTTTGATGGGAGTTCGAACAACTTTTTCTTTGCAAATTTGCCCACACCTAGAGAGAGAGGAGGAACACTTTGCTTCGCAGGTTGTGGACAGCCTCTCGTCATGTTT**GACTGTATGGATGTTCTCGC**AGTCAGCCCTGGGCAAATGCTGGATTTCTACACTCCGAGCCCGTCCTCTTGCATGCTGCAGGAGAAAGCTCTGACTGCATGCTTCAATGGACTGACACACAGGGACTGGCAACAGAGGCACAGCACTCAATCAATTGAAACACAGAGTACCAGTTCTGAAGAGATTGTACCAAGCCCACCATCCCCAATTCCACCTCCACGGGTATACAAACCTTGCTTTGTCTGTCAGGACAAGTCATCAGGCTATCACTATGGAGTCAGTGCCTGTGAAGGATGCAAGGGTTTCTTCCGGAGAAGCATCCAGAAAAATATGGTTTATACTTGTCACAGAGAAAAAAACTGTGTCATCAACAAAGTTACTAGAAATCGCTGCCAGTACTGCCGATTGCAGAAGTGCTTTGAAGTGGGCATGTCTAAAGAATCAAGCAGACAGCAGGGAGCTGACGATGTGACGTCACAATGCCGCAAAGGAGGTTCTGAAAGGAGTTTTGGCAGAGGCCTGCTGCTTCCAACTGTAAGAAATGACAGAAACAAAAAGAAAAAGGAGATGCCAAAGCAAGAATGTTCAGAAAGCTACACAATGACAGCAGAATTAGAGGATCTAATTGAAAAGATTTGCAAAGCTCACCAGGAAACCTTCCCTTCTCTTTGCCAGCTTGGGAAATATACTACAAATTCCAGTGCTGACCACCGGGTACGGCTTGACCTGGGCTTGTGGGACAAATTCAGTGAACTTGCCACAAAGTGCATTATCAAAATTGTGGAATTTGCGAAACGACTGCCTGGCTTCACGAGCTTGACCATTG**CAGATCAGATCACTCTGCTC**AAAGCTGCTTGCCTGGACATCTTGATTCTCCGAATCTGCACCCGATACACACCTGAGCAAGACACAATGACCTTCTCCGATGGCCTTACCTTGAACAGGATTCAGATGCACAATGCGGGTTTTGGCCCTCTCACTGACCTCGTCTTTACCTTTGCCAACCAACTCCTGCCACTTGAAATGGATGACACCGAGACTGGCCTTCTTAGTGCCATCTGTCTGATCTGTGGGGACCGCCAAGATCTGGAAGATGCAGAAAAGGTGGACAAATTGCAGGAACCACTTTTGGAAGCTTTAAAGATTTATATACGGAAAAGAAGACCCAATAAACCCCATATGTTCCCAAAGATATTAATGAAAATCACAGATTTGCGCAGCATCAGTGCAAAAGGTGCAGAGCGTGTCATCACACTTAAGTTGGAGATTCCTGGATCAATGCCACCCCTTATTCAAGAAATGCTGGAAAACTCAGAAGGGCATGATCCCCTTGCACCCTCAGCAAACACAAGCACAGGGAGTACTGGGAGCAGCAGTCAAGCACTAGAAGCAACTTCCAGCCCCAGTCTGTCTCCCAGTTCTGCAGCCAATGGACCAAACAGCCAGGTACCAAACACACAGTAAATGGCGCTGCAGTGACCACAACCATTCCCAAATGTGATAGTTTCTTCTCTCTTTTTTTGTTCTGAAACATCAAGGCCTGAAGGACCAGGTTGATAAGTGTAATATTCGACATTTAAACCACTGTAACTCTTGACCTAATGGACTTTTGAGTATGAAAATACTGAATAATGAACTGTGTTGGCCAGATCCTGTCCTCCTGGATTGAATGCAGTCAGTTCAAAGACTGATGCCTGGTCAAGCCCATTTGTGTGTCCACTATTGCATGCTGGGAAGAAGCTGTGGAAAATGATGTAGAACTCTGATTATTACTTTATTTTGCAGAGTTACTCCTCAATGTATTCTAAATTTCTATGCTGGAGTTTCTGGTGCTTTCTCATTGCCTTGCATATCATGTCTAAATACATACACCAAGTTATGGAACAAAGCCTAAGTACGTAAATAAGGTGAGTAATGGTCACAGGATTCTCTGGTTTAGACAATATGGGACGTTCATTATTATTCTCAATGTTACTGTTGTATTTTCTTGCATTTTCTAGTCAAACAAAGCCTGAAGGTTGGGATTTTATCCTCAGCTCACCAGCATTCAATTTTGTTAAATAAACAAGGACATATTTCCACCTCTTTTTGCAATGAGAAACAAAGTTTGATATTTAATTTTTTTTTAATACTAGCACTTTGTGCTGGTTGTAATTGTTTCCCACTGGCAGTTAGCCCACATCACCATGTTGTTTGTATGAATGTTTACAATTCACAAGCACTTAAGAAACAGATTTTTTTAAAAAATTAAAACAACTCCCTGCATACATGTTAGTCAGAACATAATATTTTTTGCGGTAGGGTACCTCCATTGCCTACTGGTTTTGTACTAGCATACAAAGCTAGTAAAAATGTTTGTTTAATTATTTCGCTGTGCAGATTCATTTCATGTTCGGCAACATATCAAGAGTTAACATTACATGCTGCATGGGTTTGCAGTAACGATAGCCTCAAAGGCACAACACTTCTCAGTGTTATTTTGTTATTTTATTTGCTTGTACATGAGCCAGTAAGTAAATGTCATCTGGTGGCTAACACACATTATATCTCTCTGCAGAAGCGTGTTCTATCGTAGATTTGTTCTTGAGTACAGTAATTTGAGTTTATTGAATCATTCATGTAGTGTCCGAAACACTAGTGGGATGTTGTTTTCTAGCTTTAGGACTGCCCTGTACTTCTACTGGCTCAGTTTGTACATTGAGATTGTTTAACAATGCTTTCTATGTTCATATACTGTTACCTTTTCCATCAAGTTTCCTGGCAAAAGAATAAAGTATATTTATTTTACGGTGTGCTCTGGACTTCATTTTTCCTGCAATATTCAACACATTTAGCCATAAAAGTTACACAGATTGGAGTAGTTGATGGCCAGAGTTAAAGAAAACGCACAGTCAACATTGTTTCCATTAATTACATTTTAAAGTAATGCGTTTAGATGTCATAACCAACATTGAACATGGATATGGACTTCAGATACATAGTTTTGGGTTTTTTTTACATAATGGAGCAATGTTTCCTCTTTAGAACAAATTTCATTTTCCAGAAGT

>RARg

GAGAGACAGGGAGAGGGAGAGAGAGCAAGAGAGAGACAGACTGCCGTGTTGGAATAACTTCAATCTTGGTTGGAGGACCCCTCGGAGGACGCGGGAGGGGCGAGATTTAAGAGATGTACCCTCCTCAAACTTGGGGCTGGTGACTGTTTGCCGGTGGCTGGAGACGGAGGGCAGGGGGGCCAGGGGGCAGGGGTTCTCTCACGCGGCCCGGACATGAACCAGGACCGCCTCTGTCCGATGGGGCACATGAGGAGTTTCCCGGCCATGATCTACTCCTTCCCCTTCGCCAGGACTCTGCGGAGCTCCCCGCCGCTCGGCGTGCTGAATACTGGGGCCTGCTACAGCAGCTACAGCAGCGGAGAATCGCAGAAGGGGGCGTCTCTGTGCTCGGTGGACACGCA**GAGTACCAGCTCGGAGGAG**ATGATACCCAGCTCCCCCTCGCCGCCGCCTCCGCCCAGGGTTTACAAACCGTGCTTCATCTGCCAGGACAAGTCGTCGGGGTATCACTACGGAGTCAGCTCATGCGAGGGATGTAAGGGCTTCTTCCGACGCAGCATTCAGAAGAACATGGTCTACACGTGTCACCGGGAAAAGAGCTGTGCTATCAACAAGGTGACTCGCAATCGCTGCCAATACTGCCGGCTGCAGAAGTGCTTCGAGGTCGGGATGTCGAAGGAATCTGTCAGGAACGATCGGAACAAGCGGAAACCGGTGAAGGAGGAGGTGCCCGAGAGATACGAGGTGACGCCGGAGGTGGAGGGTCTGATTGAGAAAGTGTGCCGAGCGCACCAGCAGACCTGTCCCTCCCTCTGTCAACTGGGCAAGTACACCACGAACTCCAGCGCTGACCACCGCGTGCAGCTGGACCTCGGGCTCTGGGACAAGTTCAGCGAGCTGGCCACCAAGTGCATCATCAAAATCGTCGAGTTCGCCAAGCGTCTGCCGGGATTCACCAGCCTGACCATTGCCGACCAGATCACCTTGCTCAAAGCCGCCTGCCTGGATATCCTGATGTTGCGAATCTGTATCCGATACACTCCCGACCAGGACACAATGACGTTTTCCGACGGCCTCACACTCAACAGGACACAGATGCACAACGCCGGGTTCGGTCCGCTCACTGATCTCCTCTTCGCCTTTGCCAATCGGTTACTGCCTCTCGAGATGGACGACACGGAGACCGGACTGCTGTGCGCGATCTGCCTCATCTGCGGAGACCGGATGGACCTGGAGGAACCAGAGAAGGTTGACCAGTTACAGGAGCCCCTCTTGGAAGCCCTGAAGGTTTACGCCAGAAAGAGGAGACCTCAAAAACCCTACATGTTCCCTCGAATGTTGATGAAGATTACTGACCT**GAGAGGAATCAGTGCCAAAG**GTGCCGAGCGAGCCATCACCCTGAAGATGGAGATTCCGGGGCCAATGCCGCCTCTGATCCGGGAGATGCTGGAGAACCCGGACGCACTGGGAACGGACGAGACGGAGAACAAGCAGGAGACGGAGGCGGAACTTTCTAGTCCAGAGGTGGTGCTGTAATTGCGAGGCGAGGTGGCCCAGACGGACACAGGAGAGGAGGGAGGAGTCAGCGGAGCAGGCGGCTGGGGGCCGGGGAAGGAGCCCTCACCATCAGCGCCTGAAAATTCCAAAATCCTCATGAGGCCACCTGGCATGTCTATCGTCTCGACGTTCCCGAGCTTCCCCCCCTCAATCGGACAGCGACCGCCCCCTTCGGACCAACAACTGTTTACATGGACTTTGGAGGGGGGTGGGGGTTGGTTTACATTGGGTGCCGGGAGGGTTGGGCGGTGGGGGGGCGAGGGGAACGGTGGGTTTGGTTGATGGGGGGGAGGCGGTTTGCGTTGCTTCGACTCGAGGGTCGATTCAGGGACATACCTCGCTCGGGGGGGGGGGCACCTGTGGGTGCTGGTGGTGGGGGGCACGTTTCTCCGATAACTGGATATTTCTTCGACATTCTCACCCCCCCCCCCCCCCCCCCC

>RXRa

CGTCAGCAGACTGTCGACCTGGGAGCGACTCAAATTATGAGAGGGTCGGACAGAACAAATCCCGGAAA**ACTGATTCCACCGTGAGGTC**AGAAGACTCTTCATCCTGTGTGAGTGTATCAATGAACTGTGAATCCATTGGAGCAGACATGAATGCACCTTCTCTTCATCCCCAGATGATGGGGTCTGGGGTCAGCCCCACCATCAGCTCCCCTGGACAGCTACATTCACCTAGAAGCACTTTGAACTCTCCAGTGAATGGACTGAGCTCTCCCTTCTCCGTTATTAGCTCTCCACTTGGACCACATACAATATCCATCCCTTCATCACACAGAATGGGCTACGGAGGGCATAGTCCGCAGATGAAACCGTCCATGAATTCAGTCAGCAGTACTGAGGATGTGAAACCGCCTTTGGGAATTAACGGTGTGCTTCAATTATCACCTCACTCCTCAAACTGTCCACTTTCCCTCACCAAACACATCTGTGCCATTTGTGGGGACAGGTCTTCAGGCAAACATTACGGTGTTTACAGCTGTGAAGGTTGTAAAGGGTTCTTCAAACGTACGGTGCGCAAAGACCTTACCTACACTTGCCGTGACAACAAGGACTGTATGATTGACAAACGCCAACGTAACCGGTGCCAATACTGCCGCTACCAGAAGTGTCTTGCCATGGGGATGAAGCGGGAAGGTATGGGCGCAGACTTCAGAGTACAAGAGGAGAGACAGCGAGGTAAGGATAGGAATGAGAACGAGGTGGAGTCGACGAGCAGTGCGAATGAAGACATGCCGGTGGAGAAGATAGTGGAGGCGGAACTGGCA**GTCGAGCCCAAGACAGAGAC**TTACATCGACTCGAATCTCAGCATGGCCAGCAGCTCGCCGAACGACCCAGTCACCAACATCTGCCAAGCAGCGGACAAGCAACTCTTCACATTGGTTGAGTGGGCCAAACGGATCCCACACTTCTCAGAGCTCCCATTGGACGACCAGGTCATTCTCTTGCGGGCAGGCTGGAATGAACTGCTGATTGCTTCATTTTCACATCGTTCCATCACCGTGAAAGATGGCATTCTGCTGGCAACTGGCCTCCATGTCCACAGGAACAGTGCACACAGCGCTGGAGTGGGGGCCATCTTCGACAGAGTTCTGACTGAGCTTGTTTCTAAAATGCGCGATATGCAAATGGACAAGACGGAGTTGGGCTGTTTGCGAGCTATCGTACTCTTCAATCCAGATGCCAAAGGTCTGTCTAATCCTGGAGAGGTGGAAGCACTCCGTGAGAAGGTGTACGCTTCGCTGGAAGGATATTGTAAACAGAAATATCCCGAGCAGCCAGGGAGGTTTGCTAAGCTCCTCTTACGATTGCCGGCCCTGCGTTCGATTGGATTGAAATGTCTCGAACATCTGTTCTTCTTCAAGCTGATTGGAGACACTCCCATTGACACTTTCCTCATGGAGATGCTGGAAGCTCCTCATCAAATATCTTAAAGCGCAGTGGAGAAGATTGCTCACCCGGGACAGACTTACATCCTTTGTAGGAGACTCAGCCTCCCCTCTGCATTTACAACCTCTTCCCAGCCCCCACGAGTATTGACACCTTGATAATCAGACAACAAAGGAATCATTTTCTCCATGTTTCTATACGCAAGAGTCTCTAGGTTGATGTCCGCTCTACCTTGAGATGTGCTGAGGTGTGTGTGTATCTGTGTGTACATTCACACTCGCACAACTCCTTGACAGTGCTCAAGGTAGCTTCTCCTTTCCTTCGATCTCCTGTTGGTTTGTGTTTTGGGTTAGTGGTGACGGGTCCGTCCTGTCCCTCTCAATCTGCATTTGTCAGTGCTGGAGATTTGGACTTTGCAGAAAACACTGGACGACCTGCAGTCCTGGTTTGAACCTTCCTGGGCTGGATGCTGTGGGGTTTGGGAGCACAAAGAGAGGTCTTGTTGATCAGACTCACTGAACCTGGACACGGCTTGGTTCTGTGCTGTTTCACCGAGGTCTCTGGACACAGCATGTTTAAGTGTTTCCAACTCATGGCATTGACTGACACTCACACCCTCGTCTGCCAACTCGATGTCCAATGCAATACATCAGTATTTGTTCATGTTTTATATTTTTCGGAGCAATAAGAGGTTTTATTTTGAAATCTCGGCGGGCAGGGCAGCTGAATGCAGTTCTTTACACAACTCTGTTCAAACCGGTGAAACCATCACTACCACAATCCAAACCCGTGTCGGGGTTCATTCCCCCTTTCCCCATGGGAACAATGGCCTTGTGCTGTTCCCATGTTGCGCCCCCTCCCACTCCGACAAATACTGGCTGGGAACGAGGCCTGACCTCGGACAATCCTTTGCTGCTAATAAATGCTTTAATTATTAACCCTTGATCGGTGACATCACGATGCTGAGGTGAGGAGAAACAGGGCTTTGTTTGCGAAATCTCTCCAAACACAGTAACTCATTTACCCCTCCCATCCTGTACACTAACTCTCTCCCAACTTGTTCCTCCTCCTCCCCAGTACCCACCTTCGACTTTGTGTACTCCATAGTTCTGTGGTTGTGCAGCGAAGGAGCTTTTTGCTCTCGCTCTCACTCCAGCTGTACCAGATGAAGGAGTGTGCAGACGGCCCTGATAGTCGCTGGTGTTTAAGCAATGCCTTGTGCTGGGGAGGGGGATTTCGCTGCCGACGATTCTGCCATTGATAACTGGGCCCACAAGACGTGTGAGGCATTTGCTCTGCTTTGTGTGACTCAGGATCCCAGTAAACTCCTCACAGCCAGCTATGGTACTCGTCTCAGTCACTTCCTTTCTTCCCTCTTCCCTCCGCACTCTCCCTCGG

>RXRb

GGAGGGACACCGATGGGCAGAGGGAGCTGGGGCTCGGGGACGATGAGGAGAAAGGGCTCGCTGCCCTTTTAATCTTGAAATCGCTGGCCCGTTGCGCCTGCATGCGGACATTTCTGCAGTTTTGGACAGAATGTGTGAGAACAGCAGAGATTGTGGGAGTCCTGACAGCTCTTCTGTGAATTCTCCACATCAGCGACGGATCATGAACAGCTCTCCGTCCATGCATACGTCAGCCATCGGGTCCTCAGTCTCGTCTTCCGTC**AACTCGCCCATCAGTATTGG**CTCGCATTTCTCCGTCATCAGCTCTTCCCTGAGCTCACCTCTTCCTTCGACCCCATCCCTGGGCTATGGGCCAGTTAGCAGCCCACAGATCAACTCCACGGTGAGCATGTTGGGTTTGCACGCCGTCAGCAGCTCCAACGACGTCCACTCGCCGCTGGACATGAACCAAGCTCTCCCTTACCCAGGCAGCTCTGCCTTATCGTCCCTGAGGCGCGTCTGTGCCATCTGTGGAGACAGGTCTTCAGGGAAGCATTACGGAGTGTACAGCTGCGAGGGCTGTAAGGGCTTCTTCAAACGGACAGTGCGCAAGGACCTCACCTACACCTGCCGAGACTGCAAGGAGTGTATGATCGATAAGCGGCAACGTAACCGGTGCCAGTACTGCCGCTACCAGAAGTGTCTGGCCACGGGTATGAAGCGTGAAGCCGTCCAAGAGGAGAGACAGAGGAGCCGAGAGAGGGGCGAAGAGGTCGAGTCCACCAGTGGGACCAACGATGAGATGCCGGTGGAGAAGATTCTGGAAGCCGAGGTGGCAGTGGAACAGAAACCGGAGCTGCAGTCCGACGGGGCAGGAAGTGAAGTGGCCTCGATGGATGCGATTTCTCAAGCACTCGCTGATGTCGAGCCCCAGGCAACAGTCATTCGCCGGGTGATTACAGCCAAAAAACCCAACGACCCGGTGACAAATATCTGCCAGGCAGCGGACAAGCAGCTCTTCACGCTGGTGGAGTGGGCAAAGCGAATCCCGCATTTCTCCGAGCTCCCGTTGGACGATCAGGTCATCCTGCT**GAGAGCAGGTTGGAATGAGC**TCCTGATCGCCTCGTTCTCGCACCGGTCGATCACAGTGAGGGATGGGATCCTGCTCGCCACCGGACTTCACGTGCACCGCAACAGCGCGCACAGCGCAGGCGTTGGAGCCATCTTTGACAGGGTTCTGACCGAACTGGTCTCCAAGATGAGAGACATGGAGATGGATAAGACAGAGCTGGGCTGCCTTCGAGCGATCATCCTCTTCAATCCCGATGCAAAGGGACTTTCAAATCCAGCAGAGGTGGAAATTCTCCGGGAGAAGGTTTACGCTTCGCTCGAATCGTACTGCAAACAGAAATATCCTGATCAGCTGGGCAGGTTTGCCAAGCTGCTCCTCCGCCTGCCTGCTCTCCGCTCTATTGGACTCAAGTGCTTGGAGCATCTATTCTTCTTCAAGCTGATCGGAGACACTCCCATCGACACTTTCCTGATGGAAATGTTGGAGGCGCCACACCAGATGTCCTGAGATGCGGGGCGCGTAGGGGAGGGGGAGGGTTGGTGGTTGGTCAAGAACGGGACAAAACAGCTGGTTGGCCACCCCCCTTCCCCCCCCATCCCTCCACCTACTCATCCCTCCACACACACCCCTCCACACACACACACACTCACCCCTCTACATACACACAAGGGTCTGAGAGGAAGCCAGGCGGACCTATGCTTGTGCTGCTGAGACAGATTGGTCAGAGTCGTGCGGCCTTTGGTTGTGTCCGTGCGACCACCCCCCCCTGTCCTGGTATGGACCTATCGATGCTGCTTGAGCCTGCCACCTTGAGCAGGCCATCTCCTGGCGGACTCGACACAGATGTGGTTGAATGGAATGAAGGTGGAGCGACTCCCGTCGGGGTTGGGGGGAGCGGTTCGCGGGTAGAGACGGCGATTCAGTTGTAAACCGAACCGATCGTCGGCCGCCATTATCCAGACTCTCCCGAGCCGCTCGCCCGACCCACTTGTAACCACGTGACTCGATCTCACTGCTTTGGTGAGCCCCGCTCGCCCCTCTACTGCGCGCTCGCTTCCCTCTTCGTCCTGCTCGTCCAGTGGGTTCGCTCGCCACCTTCCGAGGAATCCCAGTCGCTGAGGGGTCTTCACTTCTTCAGATCCGCTGGGGAGATCCTCATTCCTTTTATTTATTTAAACCAAGCCATGCTTTGGGCTCTCACAGATATCGGAGGGGGGGGGGGAGGAATGCTGGTGTGTGTGCCGGAGGTGTGGGGTGCCATTGTCGCGTGCTTACTCCTGAATACGAAGCTGCCCTGTGTGTTTCAGCGCTAACACACTGATAGTGCTGAGAATCTGTGGCTGTGTGTGCAGGCTGGCATCTGGCAGCAGGGGATAAACCTGCCGCAGCGAAACAGACAGCCCTCTGTGGCTGTGAGGGGCCCGGGGAAGCTAGTCTGATTCAGCTCCAGCCATAATCCTGAATGGCAGTATGTTAAATGTTGGGAGTGGGTGGGAGAGAGTGAAAATATTAGTAACATGCAGGCTTCAGGGACTGGACCTGAGATATATTGTTGGGATAAAATCTAGAGCTTCACTCTACATCTAACCCCCTGCTATGCCTTTCCTGGATGTGTTTGATGTGGACAGTGCTGGGGGAGGTTTACCCTGCACCTAACCCCATGCTGTACCTGTCCT

>RXRg

GGGGAAGCAGTGGGGGAGAGACGGCACCGTCTCTGGTCACTTCACACAAACACGGGAAAGCAGGAGGCTGCAGCTCCTCCTGTCTCAGCAACAACAAACTATGAAGACAGATGAGTGAATGTGGATGAGAGGCAGCAGCAGCAGCAACAACATGTACGGTCACTACCATCACTTCATGAAGCTCCCTGTCAGCTACAACAACCACTCCGCGATACAAGTGAATTCCACATCAATGAACTCCCCTCCCCCACACTCGACAGTGAACTCGATGGTTGGCCATCATTCAATGATCGGATCCTCTGCAAACGCTTCACGCTCTCTCACCACCTCCATGGCGAACCTGGCTTCCCCAGTGAATGGATTGGGATCTCCGTATCCAGTCATCACCTCATCGATAGGCTCCCCCTCGGTGTCCCTCTCATCTACTCCGGGCATTGGTTATGGACCTCTCAGCAATCCTCAGATTAATACAGTGAATAACGTCAGTAGTACGGAGGATATTAAGCCGCCACCCGGCCTGAGCGGACTTGGGAACATGA**ATTGTTACAGCCCCAACTCG**ATTGCCAAGCATATCTGTGCGATATGTGGAGACCGCTCTTCAGGAAAGCATTACGGTGTGTACAGCTGTGAGGGCTGTAAAGGCTTCTTTAAACGTACTGTGCGCAAAGATCTCACCTACACTTGTCGTGACACCAAGGACTGCATGATCGACAAGCGCCAGCGCAATCGCTGCCAATACTGCCGCTATCAGAAATGCCTGGCCACAGGAATGAAAAGAGAAGCCGTGCAGGAGGAGAGGCAGCGAGGGAAGGAGAGAAGTGACAATGAGCTAGAGCCCAGCAGCAGTGTCAATGACGACATGCCAGTGGAGAAGATTCTAGAAGCAGAACTGGCTGTGGAACCAAAGACAGAGACTTATGTGGATGCCAGTCCAGGAAATTCGTCCAACGACCCCGTCACCAACATATGCCAGGCTGCGGATAAACAGCTTTTCACCCTGGTTGAGTGGGCCAAGAGAATTCCACACTTCTCCGATCTCCCTCTCGACGATCAAGTCATTCTCCTCCGAGCAGGCTGGAACGAGCTGCTCATCGCCTCATTCTCACATCGTTCAATCTCAGTGAAGGATGGAATCCTCCTGGCCACAGGACTGCACGTCCATCGCAGCAGTGCCCACAGCGCCGGTGTGGGCTCCATATTCGACAGAGTTCTGACCGAATTAGTTTCGAAGATGAAAGACATGCAGATGGACAAGACCGAGCTGGGGTGCCTGAGAGCCATCGTTCTCTTCAATCCAGATGCTAAAGGCTTATCAAACCCAGGGGAAGTGGAATCGCTGAGGGAAAAGGTGTATGCATCGCTCGAGGCATATACAAAACACAAATACCCTGACCAACCTGGCAGGTTTGCAAAGCTGCTCCTGCGACTTCCGGCTCTCCGATCGATCGGACTCAAGTGTCTCGAGCACCTATTCT**TTTTCAAGCTGATTGGGGAC**ACCCCCATTGACACCTTCCTCATGGAGATGCTGGAGGCCCCACACCAAATGACATGATTCGATTCTCCATGCTCCCTACTTTCTAAATGTCAAATCTGTAAATATTTCGCTAATTGTGTGAATAGAATTGATATAATGTCATCCGTCTAACTTGTAAAACAACACATATTTTGTATCTTGAACAAGCACAGAACAGCAATTATTTGTTAGTTTGTACAGGGAATTTTTAAAAAACGCTTATCGGAAGAATTTTAGATTAAAATTCTATTCAGTGAAGAATAAATCAGGTTTATAAATCGGGATGCAGTACCTCTCTCACACCTCCATTCTCACAATCATTCGCTCGTTCTCATTCAACCTCTGGCCCTGGCACTTGTGAACAGGGACAGTGGTCTGAGCGAGTGCCTCTATTATAGAGCCTGTACCTCTCCATGGGCCCAATGGGAAACTGGAAATGACGCTGGGGTGATTACCTCCCTCCCGCTCCTAACAGGCAAGCGCACATTCAGAATATAGGCACTTACAAGCCAATCGTGCTGTATGAAGCCTGTTTTAAAGGGACTCTTTATCCCCCACCTGCCAAAACATAAAATGACCTTCAGATACAAGTCGTGGTTAAGCTTCTTTATTTAAAAAAATGCGTAAATCGCACTGACTACGTGTATTCCCTCTGACCCTGTGACAAATTAACTCCACTCCTTTCTCCTCTGAGGAACAATGCTCCCGATTTCAGGCCTGAAGATGAAGATTTTGACTCCTCTTCAAATTTTCCATTTTAATATTGAAGAAGATGCAGTCCTCAGTCCGAAGCGATTCTTCACCTTTTGCCCTAGACTTGACGAACATCCAGTCAATGGTCAGGTGGTCGCACAGGCTATCACAGGTAGAATGAAGGGGACTAGGGAATATTGGAAGTGATGCTTTCACACAGTAGGTCATTAAAGCATGCAGCTTGTGAGGCACAGTCTGATTCCAGAGGGAGTTGGGAAAGGATTTGAAGAGCAGGGATGTAAAAGGAAATGGGTCAGAGTGAAAGAATGGGATTGAAATCGATGGAACAAGTTGGAGAGCTAACACAAACATTGCACAATGGACAGAATGTCCTCCTGTAACCATTTTTGTTAATCTTTGGGATATGAGCATTACTGGCAAGATTAACAAATATTGTCCATCCATAATTGCCCTGAAATTATATCATGGAGATGTTTTCTAGACCATTTCAGAGGGGTGGTTAAGAGTCAACCTCATTGCTGTGGGTTTGGAGTCACAGAGGTAGGCCAGACAGGGTAAGGACGGCAGATTTCCTCTCCTATTGGAAAGAAATCATGAAAAGGAATAAGGATCAAATAATATTGAATGAATGCGGGAATATTGGATTAAACGTTAGGTTGAGTAAACTCACAACAAAGTATATTTTAATGAGGCAAATCTAGCTTGTGAGAACAGTACGGTTGACGCCCTTCACTGCAATGTGAGTTCATCGATAAGAATAGACACCCCTAAATACGGTATACAGTACACAAAAGAC
