# Supplementary figures and images for: Interference with the retinoic acid signalling pathway inhibits the initiation of teeth and caudal primary scales in the small-spotted catshark Scyliorhinus canicula
Source: PeerJ. 2023 Sep 6;11:e15896. doi: 10.7717/peerj.15896 (PMC10492535; doi:10.7717/peerj.15896)

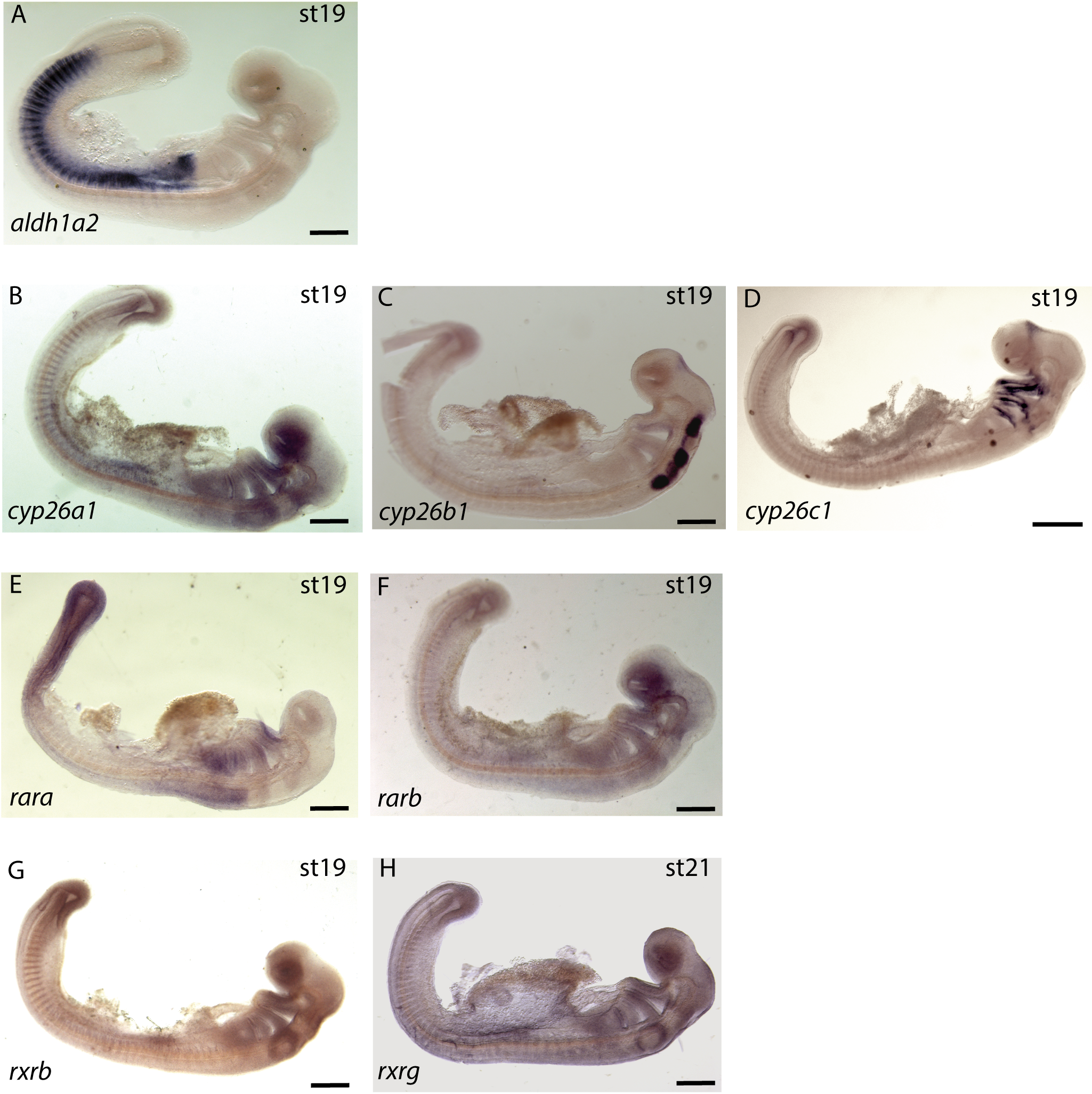

Supplement: Supplemental Information 2 — Lateral views of whole mount hybridized embryos with aldh1a2 (A), cyp26a1-c1 (B–D), rara-b (E–F) and rxrb-g (G–H) probes. For each panel, the name of the probe is indicated at the bottom left and the stage of embryo at the top right. Scale bars: 500 µm. [file peerj-11-15896-s002.png]

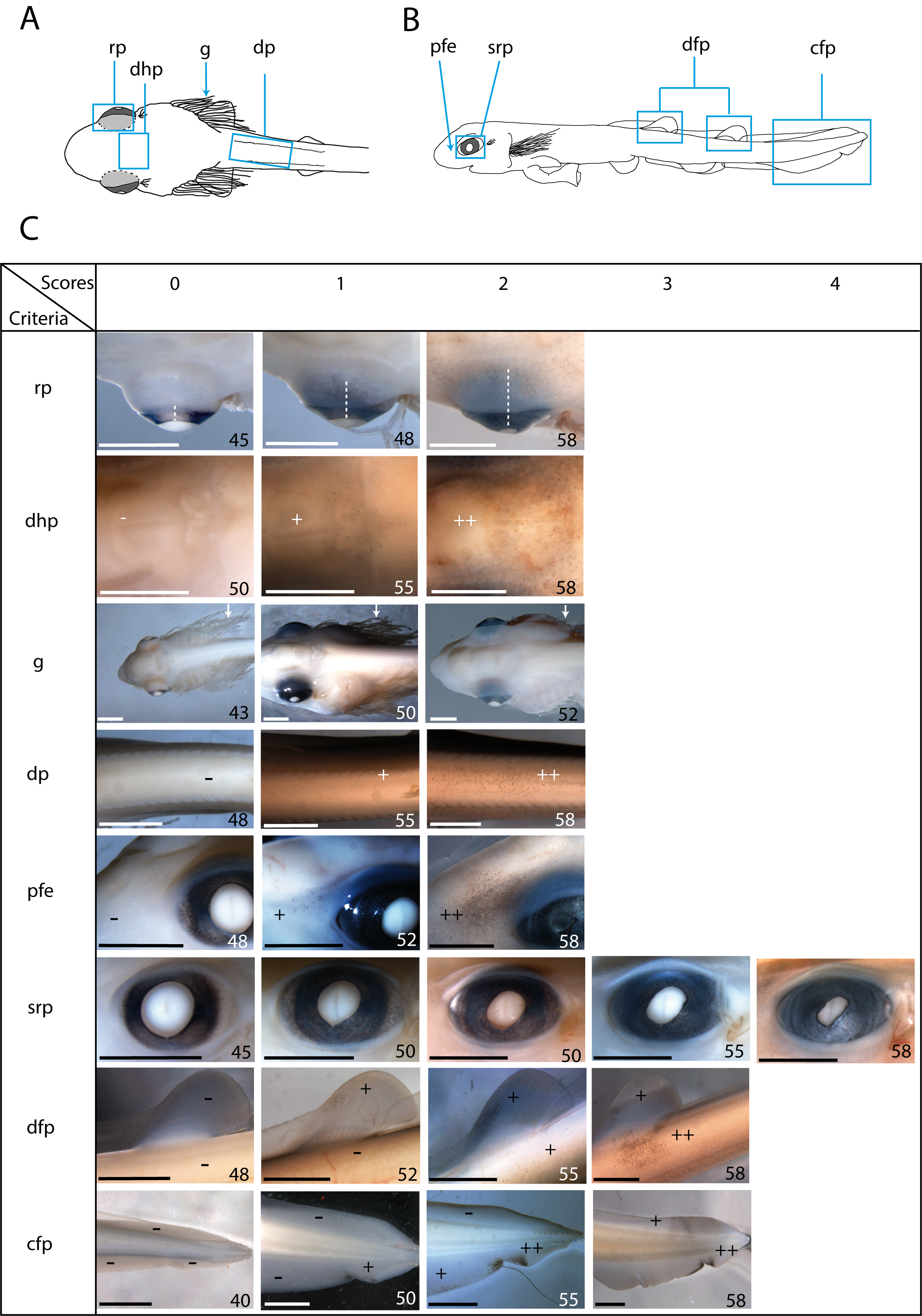

Supplement: Supplemental Information 3 — (A and B) Schematic of a dorsal (A) and lateral (B) views of a 50 mm long embryo of Scyliorhinus canicula. The boxed areas or arrows indicate our developmental criteria which correspond to regions that evolve during the growth of a 40 to 58 mm embryo. (C) Table showing the scores associated with each developmental criterion. The body length of the embryos that illustrated the scores for each criterion are indicated in mm at the right bottom of each picture. A score (0 to 4) is given according to the aspect of the observed criterion. For the pigmentation criteria (dhp, dp, pfe, dfp and cfp), the amount of pigment is assessed: − no pigment (score 0), + limited amount of pigment, ++ large amount of pigment. For retinal pigmentation in dorsal view (rp), the pigmentation area, symbolized by the white dotted lines (one third, two thirds or the entire retinal area), is considered. For the gills (g) (white arrows), the regression of their length is evaluated. The shape and pigmentation of the retina in lateral view (srp) are evaluated: crown shape of the pigmented retina (score 0), oval shape with more or less pigments in the corners (scores 1–3), opening of the retina at the lens level becoming a slit (score 4). cfp: caudal fin pigmentation, dfp: dorsal fin pigmentation, dhp: dorsal head pigmentation, dp: dorsal pigmentation, g: gills filament regression, pfe: pigmentation at the front of the eye, rp: retinal pigmentation dorsal view, srp: shape and retinal pigmentation of the eye. Scale bars: 2 mm [file peerj-11-15896-s003.png]

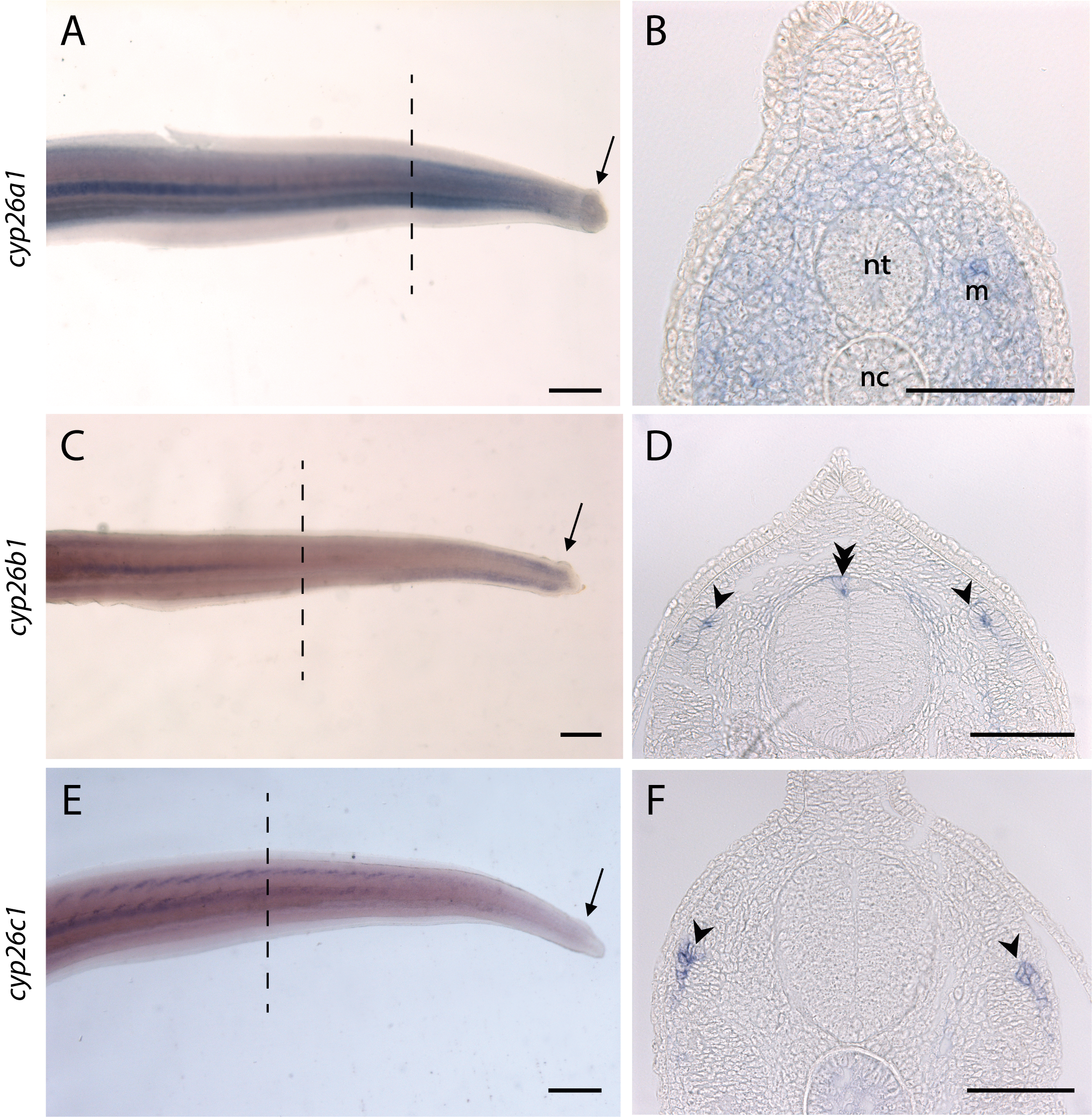

Supplement: Supplemental Information 4 — Expression profiles of cyp26a1 (A and B), cyp26b1 (C and D) and cyp26c1 (E and F) genes in whole mount hybridized tails (A, C, E) and in transversal sections of the same hybridized tails (B, D, F) of stage 29 embryos. A, C, E: anterior is left, dorsal is top. The dotted lines represent the level at which the sections are located and the arrows indicate the scale buds. B, D, F: dorsal is top. No expression of the three cyp26 genes is detected in scale buds (A, C, E). cyp26a1 was expressed in the whole mesenchyme (m) while cyp26b1 and cyp26c1 were expressed in specific regions of mesenchyme (arrowheads). Note an expression of cyp26b1 in the neural tube (nt) (double arrowhead). nc: notochord. Scale bars: 400 µm for A, C and E and 100 µm for B, D and F. [file peerj-11-15896-s004.png]

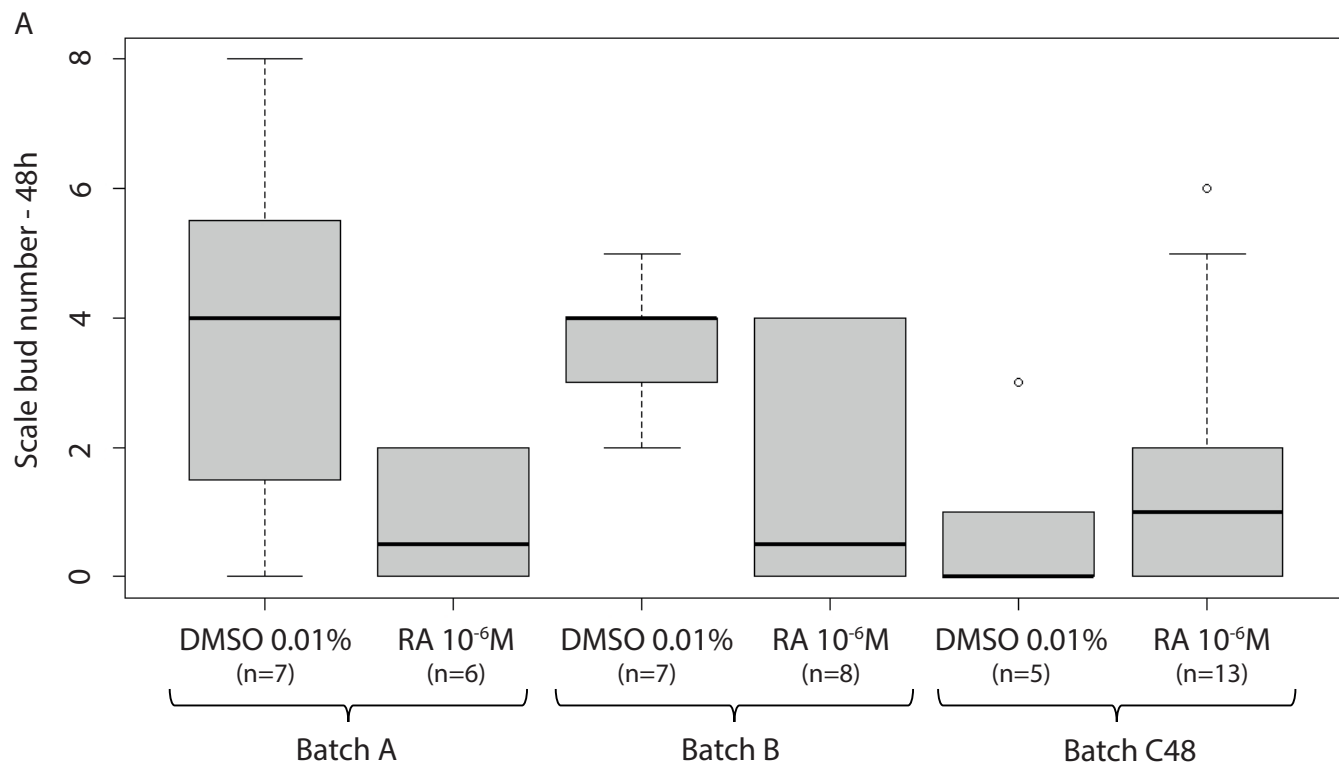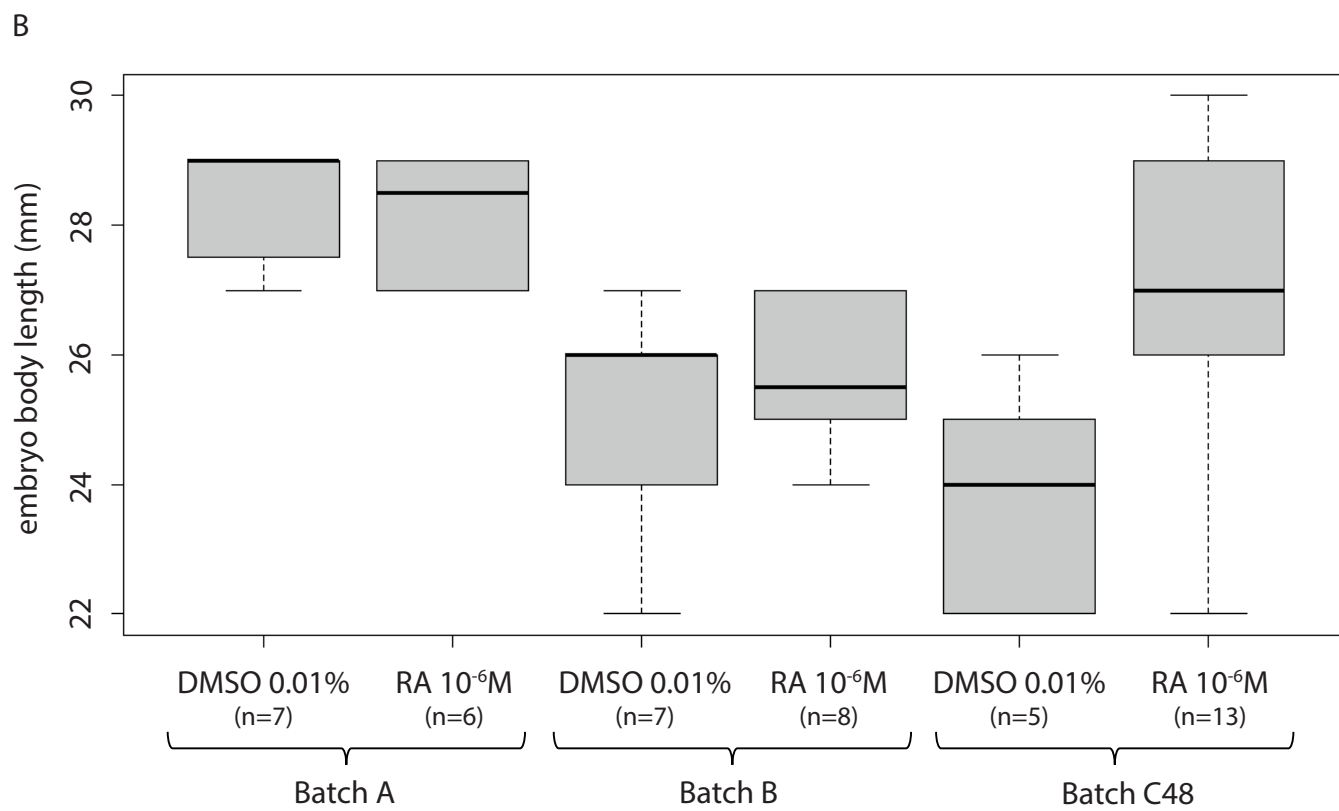

Supplement: Supplemental Information 5 — (A) Box plots representing the effect of exogenous RA on scale bud number compared to DMSO control. The black lines represent the medians, box height depicts first and third quartiles, whiskers represent the first and nine deciles. Outliers are shown as circles. Three batches (A, B and C48) of embryos were treated under the same conditions. The generalized linear model shows a significant effect of treatment (p-value = 0.038) with in most cases a decrease in the number of buds for the RA treatment, compared to their respective DMSO controls. There is alsoa significant interaction between batch and treatment (p-value = 0.034). the control embryos of batch C displaying a lower number of scales buds than control embryos of batch A. (B) Box plots representing the effect of exogenous RA on embryo body length compared to DMSO control conditions. the generalized linear model shows a significant effect of treatment (p-value = 0.011), batch (p-value = 1.48e−5) and interaction between both (p-value = 0.026). The figure shows that batch C48 behaves differently from the two other ones most probably due to the smaller length of control embryos compared to the treated ones. [file peerj-11-15896-s005.pdf]
